# The physician experience of patient to provider prejudice (PPtP)

**DOI:** 10.3389/fpubh.2024.1304107

**Published:** 2024-02-26

**Authors:** Doerthe A. Andreae, Sameer Massand, Cheryl Dellasega

**Affiliations:** ^1^Section of Allergy and Immunology, Department of Dermatology, University of Utah, Salt Lake City, UT, United States; ^2^Division of Plastic Surgery, Department of Surgery, Penn State Health, Hershey, PA, United States; ^3^Department of Nursing, College of Medicine, Pennsylvania State University, Penn State Health, Hershey, PA, United States

**Keywords:** prejudice and discrimination, retention, workforce, bias, patient outcomes

## Abstract

**Background:**

Patients can demonstrate prejudice and bias toward minoritized physicians in a destructive dynamic identified as PPtP (Patient Prejudice toward Providers). These interactions have a negative impact on the physical and mental well-being of both those who are targeted and those who witness such behaviors.

**Study purpose:**

The purpose of this study was to explore the PPtP experiences of attending physicians who identify as a minority based on race, ethnicity, citizenship status, or faith preference.

**Methods:**

Qualitative methodology was used to collect data using in-depth interviews. 15 attending physicians (8 male, 7 female, aged 33–55 years) who identified as minorities based on ethnicity, citizenship status, or faith practices were interviewed individually. Interviews were conducted using a guide validated in previous studies and content analysis was performed by two trained researchers to identify themes.

**Results:**

Five themes were identified: *A Continuum of Offenses*, *Professional Growth through Adversity, Organizational Issues, Role of Colleagues*, and *Consequences for Provision of Care*. Findings suggest that although attending physicians learned to cope with PPtP, the experience of being treated with bias negatively impacted their well-being and work performance. Attending physicians also felt that white majority medical students sometimes treated them with prejudice but expressed a commitment to protecting vulnerable trainees from PPtP.

**Conclusion:**

The experience of PPtP occurs consistently throughout a career in medicine, often beginning in the years of training and persisting into the phase of attending status. This makes it imperative to include strategies that address PPtP in order to successfully recruit and retain minoritized physicians.

## 1 Introduction

Racism in medicine is a topic of increasing concern. Over the past decade, many studies and reports have documented bias and prejudice directed toward minoritized patients (1) on both an institutional and individual basis (2). Minority patients have been found to score below average on indicative health outcome measures, receive less intensive care for common diseases and mental health, experience less stringent pain management, undergo more invasive procedures for the same conditions, and qualify for fewer organ transplants, among many other disparities (3–10). Educational and clinical interventions to address and mitigate these negative outcomes are being evaluated and implemented (11).

The reverse situation, in which patients discriminate against their physicians or nurses of minority status (race, ethnicity, citizenship status, or faith preference) is less well understood and addressed. A landmark paper by Paul-Emile et al. demonstrates that this inverse scenario is similarly exacerbated by our limited understanding and recognition of it (12). Early examination of this phenomenon compounds the already known difficulties faced by minority physicians, who are promoted at lower rates than their non-minority counterparts, awarded less grant money, and afforded a lower income (13–18). Overt and subtle forms of discrimination can occur throughout the careers of physicians, beginning in medical school and persisting into residency. Behaviors such as disparaging comments, undermining capacity, ridicule, and refusing care are examples of bias manifested toward healthcare providers (19–21).

These negative interactions between patients and providers can have many destructive downstream effects (22). Research on “difficult” patients, or patients who use physically or psychologically violent behaviors toward providers documents that such interactions cause emotional and moral distress, burnout, poor performance, and job dissatisfaction (21, 23–27). Ultimately, patients themselves are affected negatively as the quality of the patient-doctor relationship is known to influence health outcomes (28).

Concerns about an inclusive work environment can affect the recruitment of minoritized physicians, specifically at medical centers in more rural and non-diverse areas. The lack of a critical mass of minority faculty, the absence of necessary programmatic efforts, and senior leadership without diversity can influence whether a minority physician candidate accepts a position (29). Retention is also a critical—and costly—step in maintaining a diverse workforce since onboarding a physician can take up to $250,000 until they reach full clinical potential (30). Many employees who leave an organization within the first 6 months do so because of the relational environment rather than the actual work demands (31).

Maintaining a work environment within which minoritized physicians can securely and safely work for long periods while providing optimal care for patients requires organizational commitment to addressing PPtP (32). Efforts to diversify medicine within specialties have been implemented on national and individual organizational levels with varying success. Effective initiatives have focused on recruitment measures, while others seek to balance the disparate incomes, promotions, and grant awards that negatively affect minority physicians (33–35). Few, however, address PPtP, which can have an equally profound effect on the well-being and retention of minority physicians.

We have previously delineated the PPtP experiences of resident physicians and nurses (unpublished data) and seek to complement that research by studying the attending physician community. Progress in retaining a diverse workforce will provide optimal care for a progressively more diverse patient population while affording attending physicians a safe, fruitful, and healthy work environment.

### 1.1 Study aim

The purpose of this study was to examine the experiences of attending physicians who have been subjected to PPtP.

## 2 Methods

The consolidated Criteria for Reporting Qualitative Research (COREQ) was used to ensure objectivity and fidelity of methods used to conduct this study (36). The research team consisted of one faculty researcher with expertise in qualitative research (CD), one physician researcher who immigrated to the US after medical school (DAA), and four medical students and one resident (SM) from diverse backgrounds.

This study was conducted and completed while all members of the research team where at Penn State. Once IRB approval from the Penn State IRB to conduct the study was received, an invitation to participate was disseminated through emails to Department chairs at Penn State, online via the Junior Faculty Development Program at Penn State and posted on announcement boards throughout the hospital as well as word-of-mouth. All attending physicians at Penn State Health meeting the inclusion criteria of self-identification as a minority based on ethnicity, citizenship status, country of medical school, or faith practices were invited to contact the investigator via email if they were interested in participating.

This study was exempt from after potential interviewees received a summary explanation of the research, a research assistant arranged to meet with them at a convenient time and place that ensured privacy on the end of the interviewer. Arranging for a quiet and private place was encouraged also for the interviewee when scheduling the interview. All interviews were conducted via Zoom using the PPtP Interview Guide validated in previous studies (see Supplementary material). Where appropriate, prompts were used to elaborate on the responses. Interviews lasted 60 min on average. Each physician was interviewed once, with no repeat interviews offered. Four medical students functioned as paid research assistants who conducted all interviews. Each had received a four-hour training on qualitative methods and successfully completed a pilot interview with a physician of minority background under the supervision of the investigators. Periodic audits of completed interviews were conducted by the investigators to assure continued fidelity.

The first 15 participants who expressed interest completed interviews, with saturation being reached after the 14th interview, with an additional interview conducted for veracity (Data saturation was defined as the point when additional interviews did not render additional information). Our sample size is within the reported range of a recent meta-analysis describing that within 9–17 interviews (mean12-13), most themes are captured (37).

Interviews were recorded and stored in a secure file (stored in Penn State box as apple audio files) prior to transcription. The transcription was performed by a paid transcriptionist within the Penn State system. The audio files were de-identified prior to transcription. After transcribing and cleaning, transcripts were reviewed independently by the investigators. With an iterative process that compared each transcript across and within interviewees, recurring words, phrases, and concepts were noted. The investigators (CD and DAA) then met to compare results and merge them into themes. The Kappa coefficient of agreement between the researchers was 0.88. An independent reviewer (SM) examined the PPtP Interview Guide questions and content relevant to PPtP, then compared the themes and exemplar statements for consistency and validity. After data extraction and theme identification, the investigators reviewed the interviews to identify sample quotes.

## 3 Results

The 15 interviewees (8 male, 7 female, aged 33–55 years) represented a diverse group in relation to ethnicity/immigration status, age, gender, and type of practice. All were employed by the same academic healthcare system, but some of the experiences they described occurred at other institutions and/or during training. For demographics, see Table 1. Exemplar statements from the transcripts are contained in Table 2. Five themes were identified based on the analysis of the researchers. *A Continuum of Offenses*, *Professional Growth through Adversity, Organizational Issues, Role of Colleagues*, and *Consequences for Provision of Care*.

**Table 1:** Participant demographics.

Characteristic	Value
*Gender*	
Male	8
Female	7
*Age (in years)*	
Mean	39.5
Range	33–55
*Area of practice*	
Surgical	2
Medical	13
*Patient age*	
Pediatric only	2
Adult only	7
Both	6
*Self-identified minority status*	
South Asian/Indian	5
Asian	3
Black	4
Hispanic	1
Sikh	1
Pakistani	1
*Self-identified IMG status*	
IMG	5
US medical school	10
*Country of birth*	
Non-US	7
US	8
*Country of citizenship*	
US only	8
Foreign only	2
Dual (US/foreign)	5
*Country of undergraduate*	
US	10
Foreign	5
*Country of medical school*	
US	10
Foreign	5
*Other post graduate training*	MPH, MS, MS, MBA, PhD

**Table 2:** Exemplar statements from thematic analysis.

Theme	Exemplars
*A continuum of aggression*	“I would say I felt like sometimes you do not hear the words but it’s the body language. I do pay special attention to people’s body language and how they communicate.” (Participant 1, p. 2)
	“[S]o they say, well, I do not want this provider because he has a foreign medical degree…and they are clearly prejudicing…” (Participant 1, p. 5)
	“If the physician is a minority the patients tend to talk to the medical student and not the attending physicians, um, just kind of the way that, I think um especially female physicians tend to have that problem.” (Participant 5, p. 3)
	“I feel like the darker you are, the more prejudice there is.” (Participant 8, p. 8)
	“[T]hey used a few choice words at me and they said something along the lines of, “Go back to where you came from,” and then hung up on me.” (Participant 9, p. 1)
	“And there was a child in the room that said, ‘I have a question.’ And I was like, ‘Yes, what is your question?’ The child said, ‘Well when is the doctor going to come?’ and this is a child of color … so I said, ‘Well I’m actually the doctor.’ And with the straightest face, without pause, without hesitation, the child said, ‘You cannot be, because you are not. You’re black.’” (Participant 13, p. 3)
	“[S]o they scheduled and then when they found out that (resident) physician was actually Muslim, they were like, ‘Oh we do not want to … We do not want to see anybody like that.’ And like, I had to intervene there and talk to the patient, saying like, … ‘You either see them or you can see care elsewhere.’” (Participant 14, p. 4)
	“[T]he biggest one would be like, almost the veiled compliment of, ‘Oh you speak English really well,’ like with an assumption of ‘Oh, you are from somewhere far away…so it’s kind of like a micro thing.” (Participant 14, p. 1)
	“But when I first got here…there was a lot of like, ‘eye-stretching’.” (Participant 15, p. 1)
*Professional growth through adversity*	“So, I have learned in a way to look beyond not the reaction but what was the cause of the reaction.” (Participant 1, p. 6)
	“There are times when it’s curiosity … and I do not know if I’m reading too much into it either, because if this is coming from someone who is an immigrant themselves, I assume it’s curiosity.” (Participant 2, p. 4)
	“I’m more comfortable, um, explaining to patients of what my boundaries are, where, what the expectations are and where, what our, what our goals are and where we are in the patient-doctor relationship.” (Participant 5, p. 7)
	“So, the more experienced I’ve become, the more I’ve worked with, um, others both in my professional and personal life, the more I’ve evolved in terms of, um, responding to these types of situations.” (Participant 9, p. 4)
	“I think the main thing I tell myself is, ‘Do not take it personally.’ Maintain a professional relationship and respectful and um, you know with time, people will know who you are, what kind of doctor you are… Model yourself in a way that you are yourself. Hopefully. I try to educate myself not to be judgmental, not to have the bias, and hopefully the patient can see that too. I think through role modeling, through setting the example. That’s my philosophy.” (Participant 10, p. 5–6)
	“I feel like I have gotten better because I have ‘tools in my toolbox’ for how to address, um, a racist or a prejudiced patient because of what I’ve learned.” (Participant 15, p. 8)
	“[E]arlier I used to get mad or upset or annoyed about it. Now I’m, you know, it’s kind of like water on a duck, it kind of just rolls off me, unless it’s just egregious. Right? And even if it’s egregious, like it’s I’m annoyed or made for maybe 5 or 10 min and then I move on.” (Participant 12, p. 4)
	“So before, where I did not feel comfortable, I may not have felt comfortable speaking up because I was a resident who was lowest on the totem pole and of all the things in medicine the one thing, I probably do not like about it most is this concept of hierarchy…where the intern is the lowest person on the totem pole so they feel the most vulnerable, they feel the most unprotected and then they are the ones who are going kind of through the most changes, um, trying to establish themselves. Um, so you know when I encountered something, when I was an intern, I-I ignored it…I never reported anything to anyone, not once…. so when I –when I encountered them as a resident, it probably made me angrier sooner or faster or longer. Now, I’m a bit-I’m a bit dismissive about that. Dismissive just because of repeated, you know repeated offensives, right?” (Participant 12, p. 13)
	“[W]hen that happens you just try to deliver the best care that you can because that’s what we are trained to do and that’s what we want to do. I mean, I’m not going to let you affect my character. I’m going to treat you like I would treat my mom.” (Participant 15, p.3)
*Organizational interventions/issues*	“Every institute should have their own policies about this.” (Participant 1, p. 8)
	“Um so long as we are within this model of patients are, we are rating providers like an Uber driver um and I mean, the issue is that there should be reciprocity, right?…If patients also had a you know, a point system where they would not have a physician if they had a certain star system or whatever in the same way as providers are then they would behave themselves in a more appropriate way, however that’s not something we would ever want to encourage…I’m just pointing out how flawed the system is.” (Participant 5, p. 8–9)
	“[W]hen they, patients have unpleasant experience with the physician provider or our physician provider they go to patient care representatives and they inform them and the patient representatives come back to the provider and they get information too and try and sort out what the issues are and address the issue…so I think there should be something like, I’m not, um, I do not know if he can go and report that to the patient care…but like instances like bias or prejudices, I do not know if there’s a means for us to go to somebody in the system and say, ‘Hey, this is what I had experienced and is there any way you can speak or communicate with the patient and say that this is not appropriate.” (Participant 6, p. 6)
	“I think letting them know we cannot have them [prejudiced patients] here anymore, which seems very extreme but I think it supports, um supports your providers rather than the patients.” (Participant 7, p. 5)
	“[I]n our faculty meetings we have been having like, there’s been a lot of like, um, kind of how to be more like, proactive about these situations and how to be more of an upstander and so in some of those conversations there’s been a few strategies and one of them was like basically just trying to just acknowledge without supporting it.” (Participant 14, p. 1)
	“Um I think the biggest, like comes to like being a unified front as an office. So, I think like enforcing policy. Like we have great policies written down, but I think, you know, from a scheduling level to a if they call in and mispronounce someone’s name, like correcting them…kind of like nipping those microaggressions in the bud, but I think as a united front.” (Participant 14, p. 9)
	“And then, you get evaluated on your patient satisfaction scores and I think you have probably already seen the research that black and brown people have lower scores generally speaking…is it just because we are worse at our jobs collectively? Doubtful.” (Participant 15, p. 6)
*Role of colleagues: positive or negative*	“I’m not saying it’s always a problem with the patient, sometimes it could be the physician prejudice and bias as well.” (Participant 6, p. 6)
	“And, um, so work as a team, this way you do not feel lonely. I truly feel I’m not lonely. I truly feel like I have the support, you know my nurses, my front desk, and my medical director and ah, we all work together very well and we respect each other very well and ah, help each other. At least I perceive it that way.” (Participant 8, p. 9)
	“I’ve had experiences being treated by both attendings and by patients, ah, as being like, less smart or less confident, or less capable because I think being a woman and being a colored woman.” (Participant 8, p. 10)
	“It’s really good to have, to work in an environment that you feel like you are, you know, colleagues to support you and can talk to you and you can talk to them too.” (Participant 10, p. 7)
	“Some of our colleagues…still believe that because you sound different, because you look different, ah, because you were not trained as well, you are less competent than them until you sit down and test…then you realize that they were completely wrong… so my final word is like, it starts here at work…the change starts here.” (Participant 11, p. 8)
	“I’ve had patients say that to my face, ‘I do not want you as my doctor.’ See, I’ve had nurses text me that like, ‘Oh the so-and-so patient says they do not want you to come back into the room’ again.’ Right? If I’m the only provider then it’s not an option for them, it just is not. That’s the reality of how healthcare works in the hospital.” (Participant 12, p. 11)
	“So, I feel like the policy is great, but the execution is maybe not perfect because I think everyone’s natural tendency is to be like non-confrontational, so if a MOA at the front is like, getting a phone call they are more likely to say, ‘Oh so and so has a slot here, just go see them’…they do not like, make a stand because that’s more work.” (Participant 14, p. 5)
*Consequences for provision of care*	[Recalling an incident in medical school where a patient was openly racist]: “And they brought security in and escorted her out. That did change our interaction and the care she got because she got escorted out of the clinic.” (Participant 2, p. 7)
	“If you have a bad patient/doctor relationship, does that ultimately affect something in the patient interaction? And I would say yes, because a lot of medicine actually has nothing to do the actual treatments that would do. It’s actually the patient interaction.” (Participant 4, p. 7)
	“And so I think it’s easy to separate and still give good care. Maybe not the most personal care because you tend to then wall off a little bit and not say oh how’s your family or how’s your…the silly things you ask about especially in pediatrics…you just tend to be a little more quiet.” (Participant 7, p. 3)
	“And when I see that patient back, I know, that I do not feel as, I like them less naturally…I probably, to some level, do not spend as much time like following up with them or investing in their care, um, just because I did not appreciate how they treated, you know, our coworkers…I think that’s sort of natural…, the more you, the patients who are sort of nicer and respect the time you give them… you are going to give them more time.” (Participant 8, p. 5)
	“I mean, I think, it’s something that a lot of providers are used to, or you know, recognize that it’s just part of their job, and trying to separate like emotional reactions or personal feelings making sound medical decisions, so it may have led to like less lab work with the patient, maybe less frequent communication, um maybe, but I think in-in general, like in terms of the quality of medical care and providing the right diagnosis, or testing or treatment, I have not witnessed that being different.” (Participant 8, p. 6)
	“[S]o sometimes it’s being extra nice or taking extra time too with that patient or doing maybe you order more tests than you normally would because you do not want to displease this patient.” (Participant 12, p. 8)
	“But medically speaking I-medically speaking, I-I, if anything, medically speaking I’m actually, I try to be more, um, conscience-conscious of my own, ah, feelings towards that patient. Make sure that I’m doing what, I’m doing my due diligence right? And I’m not simply doing something because I, one, I’m dismissive of what they are saying, or because I do not like them.” (Participant 12, p. 9)
	“So, you have to, so I cannot say that it’s not going to affect some kind of care, if all you are feeling is disrespect for that patient. Does that mean you want to get them out of the hospital faster? It might. Does it mean you want to hand him off sooner? …And you-and you already know it. Is it-there has been tons of data, tons of literature, it shows the more ah, physician hand-offs, the more patient hand-offs you have had between physicians, the more families fall through the cracks, the more mistakes happen, the more bad outcomes, right? So even though we are handing these-we are handing these difficult patients off, that means information is inexplicably being lost, it’s being, information is just being lost, right? A new provider cannot know everything that happens with that patient prior to them taking it over. They just cannot. They can read as much as they can, but data’s going to fall through the cracks. Slight things: little conversations; little phone calls you have had with other consultants. So, like, so definitely at some level patient–patient care is being affected. (Participant 13, p. 3)
	“[Y]ou have to take that unfortunate truth and place it aside to deliver the care that you have been trained to deliver. Um, so I do not think it is impacted. Ah, for me, at least in my few instances it has not impacted what I, the caring I provide. I would still advocate for those patients the way I did regardless of the surrounding circumstances or situations.” (Participant 15, p. 3)
	“Um, but we have to admit that-that happens, and so, when that happens, you just try to deliver the best care that you can because that what’s we are trained to do and that’s what we want to do. I mean, I’m not gonna—I’m not going to let you affect my character. I’m going to treat you like I would treat my mom.” (Participant 15, p. 3)

### 3.1 Continuum of offenses

Situations in which the provider was the target of some form of PPtP from patients and their families were described by most interviewees, with the emotional impact of these interactions differing. For example, remarks about a physician’s ability to speak English happened so frequently that some chose to laugh it off. One told a patient: “If I could not speak [English] well, it would be a condemnation for the American public school system, since that’s where I learned” (Participant 2, p. 5).

At other times, the reaction to the aggression went deeper, especially when physicians felt they were viewed as “less than” their non-minoritized peers. Physicians reported that refusal of care from a patient felt specifically hurtful. In one situation where a parent was doubting the physician’s capabilities because of ethnicity, the physician said,

I had to call him out on it, and say like, “Sir…what are your concerns?” He’s like, “I just, you know, I do not appreciate that foreigners are like, doing this.” And I was like, “Well you know I grew up in [US state], like, you know, I do not really consider myself a foreigner.” (Participant 14, p. 2–3).

### 3.2 Professional growth through adversity

There was consensus among interviewees that PPtP happened more frequently in medical school and residency, but by the end of their training, many physicians reflected that part of what they had learned was strategies for how to react. One interviewee explained:

[Y]es, I’ve matured and changed and experienced that, but that coat of armor has built up. The skills of talking with patients ha[ve] built up. The skills of upstanding and by-standing and being an advocate, those skills have developed over the years, so it’s yes, I can take a lot more than I once could…you develop some comfort level but you are not always comfortable, right? It’s like this concept of being comfortable with the uncomfortable, ah, in many instances. (Participant 12, p. 14).

Interviewees reported that after completing their training, there was a feeling of security and confidence in their skills; thus, they felt less personally offended when PPtP occurred. Sometimes this was a deliberate stance and, at others, almost automatic as physicians refined their technological expertise.

The recognition that vulnerability was most evident in the early stages of training was illustrated by one interviewee this way: “[T]he intern is the lowest person on the totem pole so they feel the most vulnerable, they feel the most unprotected and then they are the ones who are going kind of through the most” (Participant 12, p. 14). This led interviewees to feel a special protectiveness toward those who were still trainees, especially if they came from minoritized backgrounds.

### 3.3 Organizational interventions/issues

While there was agreement that the hospital as an institution and employer held some responsibility for resolving PPtP, physicians recognized that certain parts of their work environment led them to believe there was no easy resolution or remediation. Noting that medicine is increasingly a “consumer-focused” profession, the anonymous power a patient can have over a physician via feedback and evaluations is tremendous and limits corrections, according to interviewees. Often manifested by scores on satisfaction surveys or feedback to administration, this contributed to burnout and anxiety in addition to feelings of rejection. Regardless of the legitimacy of a complaint, once it was entered as a patient comment, the physician perceived a type of “double hit”: bias from the patient during the encounter and later negative consequences from the system because of the low satisfaction score or negative comment. The fallout of organizations failing to take action to address PPtP was significant. Said one physician:

I would say [PPtP] has deeply impacted my job satisfaction. I still love working here…but I think, I do miss, um, having a diverse population…I did not expect to feel that way but it’s also harder to relate to someone who does not appreciate different cultures or different personalities or different backgrounds. (Participant 8, p.9).

Many saw it as their employer’s imperative to create a workplace that welcomed diversity and positively acknowledged attempts by the institution to do so. An interviewee said,

So, if I’m Hospital A and Hospital A needs to be comfortable explaining to people of the community A lives in that we hire and employ people who are quite skilled at providing their care and reassure them that even though the person may look different than them, the standard that we have in place for who cares for the people in our community is unrivaled and because of that, we ask these people to come in and serve and care for people who might be ill. Ask them to respect their service, and this is what respecting their service looks like. And having that be a clear part of the mission, the vision and values of Hospital A. (Participant 13, p. 4).

### 3.4 Role of colleagues: positive or negative

Although participants were not explicitly asked about prejudice and bias originating from coworkers or other individuals in their work environment, when describing experiences with patients, this influence was frequently commented on. Said one,

[I]t was a nurse who—it was their patient, their name was on the chart to call for questions, they were the senior resident, but the nurse decided to call the junior resident…the junior resident was not black. So, then the junior resident had to call the senior resident to help…. when they asked [the nurse] why they did not call them, they were very quiet…so there was a feeling of bias against calling the senior resident. (Participant 13, p. 1).

The interviewee reported that in this situation, bias against the resident was not addressed because it was difficult to know how to do so. When bias came from team members, there was significant frustration:

Some of our colleagues…still believe that because you sound different, because you look different, ah, because you were not trained as well, you are less competent than them until you sit down and test…then you realize that they were completely wrong… [and] …some of them feel like they have to diminish you to be able to be seen as a good physician. (Participant 11, p. 8).

A supportive work environment was described as a very important factor in dealing with the daily impacts of PPtP; specifically. This often includes colleagues who were understanding and provided safe spaces to discuss experiences. One interviewee commented that: “It’s really good to have, to work in an environment that you feel like you are, you know, colleagues to support you and can talk to you and you can talk to them too” (Participant 10, p. 7). This positive culture within teams was not limited to direct colleagues, as demonstrated in this comment:

I truly feel like I have the support, you know my nurses, my front desk, and my medical director and ah, we all work together very well and we respect each other very well and ah, help each other. At least I perceive it that way. (Participant 8, p. 9).

These physicians reported that the actions of both supervisors (administrators) and colleagues (coworkers) had an influence on the work environment and could contribute to positive evaluations of the interviewee (and therefore bonuses). Direct support from colleagues or supervisors seemed to have a more effective strategy than institutional policies, which were felt to be inconsistently implemented and often ignored.

### 3.5 Consequences for provision of care

PPtP was shown to impact not only the affected physicians but also had a negative effect on patient care, as evidenced by this comment about a patient who exhibited prejudice toward the physician: “I probably, to some level, do not spend as much time like following up with them or investing in their care” (Participant 7, p. 3). Trained to deliver quality patient care in a compassionate way, providers on the receiving end of PPtP found themselves in an ethically challenging position due to conflict between providing optimal care while simultaneously managing the emotional effect of bias from the patient. Ultimately, they persevered, as demonstrated by this comment:

[W]hen that happens, you just try to deliver the best care that you can because that what’s we are trained to do and that’s what we want to do. I mean, I’m not gonna-I’m not going to let you affect my character. I’m going to treat you like I would treat my mom. (Participant 15, p. 3).

Even though PPtP became a “norm” and interviewees felt like they became used to it, one said: “I think, it’s something that a lot of providers are used to, or you know, recognize that it’s just part of their job, and try to separate emotional reactions or personal feelings making sound medical decisions” (Participant 8, p. 5).

The reported effect on patient care ranged from overcompensation, fractured communication, and less diligent care. Some physicians described practicing medicine with extra caution after a patient addressed them with prejudice. “[S]o sometimes it’s being extra nice or taking extra time too with that patient or doing maybe you order more tests than you normally would because you do not want to displease this patient” (Participant 8, p. 6).

## 4 Limitations

This study was limited by the use of one institution to recruit participants, although many interviewees drew on experiences at other hospitals. While qualitative methodology limits generalizability, current studies using quantitative measures with a larger population are in progress.

## 5 Discussion

The PPtP experiences of attending physicians described in this study are consistent with behaviors identified in our previous studies. They include both overt actions such as refusing care or making negative comments as well as covert gestures such as “side-eyeing” or body language. As with our previous work, female residents or physicians felt more targeted, especially when they were mistaken for a nurse or received sexist comments on their appearance (22).

For most interviewees, experience led to role security and the development of specific proactive responses to PPtP. Often, this involved attempting to make meaning of the behaviors in ways that allowed physicians to continue providing care, such as reasoning that the person was ill and not their best self. At other times, this involved giving patients the benefit of the doubt or using standard responses to PPtP (validating their credentials, seeking outside help) so the therapeutic relationship would not be fractured. Others even adopted an attitude of forgiveness, rationalizing that even white physicians could be on the receiving end of unpleasant and rude behavior from individuals who were sick. Additional approaches included setting boundaries, humor, tolerating, and ignoring. Still, the experience of PPtP was coped with at the cost of spontaneity, connection, and professional well-being.

Despite their resilience in responding to PPtP, attending physicians were still concerned about the ethics of such challenging situations, which often required them to provide care for patients who treated them with disrespect and even outright rejection. Also, the impact of such interactions on patient satisfaction scores was worrisome, and negative comments from patients influenced job satisfaction and might ultimately affect promotions or bonuses.

The relatively higher status, self-confidence, and clinical skill that came after the initial phases of training provided not only the ability to respond better for oneself but a feeling of protectiveness towards trainees who found themselves in these situations. However, being cast in the role of educator had a threatening as well as a motiving impact. Teaching students who were “mostly white” caused some to question their abilities, especially when they had an accent or were educated outside the US.

## 6 Conclusion

Our studies have helped highlight a multitude of problems arising from PPtP in healthcare settings. Some hospital systems have implemented a zero-tolerance policy toward bias and prejudice against members of the healthcare system taking a clear stance. However, these policies are often hard to implement in real life. As one participant said, “Policies do not take away prejudice.” More research on the effectiveness of organizational responses to PPtP as well as larger interdisciplinary and quantitative studies are needed to better address and resolve this destructive dynamic. The multi-institutional intervention study by Kalet et al. (32) offers promise in this area.

Many of the PPtP situations described by our interviewees were microaggressions, meaning bystanders who have never been on the receiving end of these biases may not recognize when they occur or consider them a form of prejudice. The affected provider then can feel not only disrespected but also isolated in their experience. At the same time, bystanders who witness more overt forms of rejection, such as belittling or humiliation, describe a sense of helplessness in reacting appropriately (22). Depending on the status or experience of the bystanders, confronting the patient and immediate response to the situation might be difficult. However, acknowledgment of the situation from non-affected team members has been reported to decrease the feeling of isolation and increase team coherence (38). Open discussion of actual or hypothetical PPtP situations within teams has also been reported to increase the chance of bystanders becoming upstanders in future situations (39).

Discord and disrespect within healthcare teams can lead to miscommunication, increased stress, and ultimately worse patient outcomes (40). As reported in our study, rejection of healthcare team members based on their race, ethnicity, immigration status, or religious affiliation by patients and/or their families can lead to diminished motivation to provide care, a sense of rejection, feelings of being let down by the employer, and difficulties with coworkers—all of which can lead to burnout and turnovers.

There is value in being proactive and persistent. In both basic and graduate medical educational settings, PPtP should be openly discussed before it occurs, not reported when there are problems. Strategies for responding should be shared and individualized. As well, all attending physicians should begin their career with an awareness of what PPtP is, how it impacts both providers and patients, and what responses can be effectively used to react to it. Our interviewees were resilient and creative in addressing their experiences of patient prejudice and bias—now they and others like them should be given a voice to share and empower others with their wisdom.

## Data availability statement

The raw data supporting the conclusions of this article will be made available by the authors, without undue reservation.

## Ethics statement

The studies involving humans were approved by the Penn State Health Review Board and considered exempt STUDY00016826. Written informed consent for participation was not required for this study in accordance with the national legislation and the institutional requirements. The studies were conducted in accordance with the local legislation and institutional requirements. The ethics committee/institutional review board waived the requirement of written informed consent for participation from the participants or the participants' legal guardians/next of kin because the study was considered exempt.

## Author contributions

DAA: Conceptualization, Funding acquisition, Investigation, Methodology, Resources, Writing – original draft, Writing – review & editing. SM: Writing – original draft, Writing – review & editing. CD: Writing – original draft, Writing – review & editing, Conceptualization, Investigation, Methodology, Supervision.
