# Supplementary material for: The physician experience of patient to provider prejudice (PPtP)
Source: Front Public Health. 2024 Feb 26;12:1304107. doi: 10.3389/fpubh.2024.1304107 (PMC10925775; doi:10.3389/fpubh.2024.1304107)
Supplement: Supplementary file 1 [file Table_1.DOCX]

Supplementary Material

# Supplementary Data

**Appendix One**

Date of Interview _/_/202_

For the purpose of this study, we're considering Patient Prejudice Toward Providers (PPTP) to be any negative behavior related to the perceived ethnicity or immigrant status of the provider.

1. **Based on that definition, could you start by describing some times when you saw PPTP between one or more patients and any kind of provider? (This could be behaviors, words, or side comments to other people).**
   1. who was involved
   2. what was said or done
   3. how the incident was handled
      1. By you
      2. By others
   4. how often might you have observed in a typical week
      1. Increasing or decreasing
   5. what explicitly made you think this was this type of prejudice
2. **Next, please describe some times when you were involved as the target of PPTP. Talk about a time when you had any kind of negative interaction or assumptions (positive or negative) that were made towards you. Have you ever felt targeted because of the way people address your background? That is patient words, behaviors, or side comments to other people. (e.g. Where are you from? I don’t trust you because x, y, z reasons).**
   1. during which stage of your career did each incident occurred (medical student, intern, resident, fellow, attending)
   2. who was involved
   3. what was said or done
   4. how the incident was handled
   5. what explicitly made you think this was this type of prejudice
3. **When these kinds of situations occurred, what made them seem like prejudice or bias rather than icebreakers or curiosity? (For example, being asked “Where are you from?” could be a conversation starter or not.)**
   1. words the patient(s) used
   2. body language
   3. other
4. **How did you react to the situation?**
   1. if other people were present, how did they react?
   2. describe anything else that happened after the situation
   3. provider transferred care
   4. patient refused care
   5. other
5. **How, if at all, did PPTP effected the care you have seen provided in any of the situations you’ve witnessed?**
6. **How, if at all, did PPTP effected the care you have provided in any of the situations you’ve witnessed?**
7. **Do you feel that there are certain patients more inclined to PPTP?**
8. **Over time, how has your response to PPTP changed, if at all?**
   1. what strategies have you used to respond

*Probe if provider has given benefit of the doubt:* You are very generous in giving people the benefit of the doubt, how have your past experiences given you the ability to do this?

1. **In your opinion, what kinds of things can be done to address patient to provider prejudice and/or bias?**
   1. Have you ever felt you were expected to tolerate behavior like that?
2. **What is this impact on your job satisfaction?**
3. **What else would like to add about PPTP? Is there anything that we haven’t covered?**

**Any other comments in general?**

Thank you for your participation!

**Other probes:**

Say more about [that]?

Could you explain [that] in more detail?

When you said [that] did you mean….

If this comes up use these follow up questions

- What minority statuses do you identify with (give examples)
- What minority status do you think people perceived

**
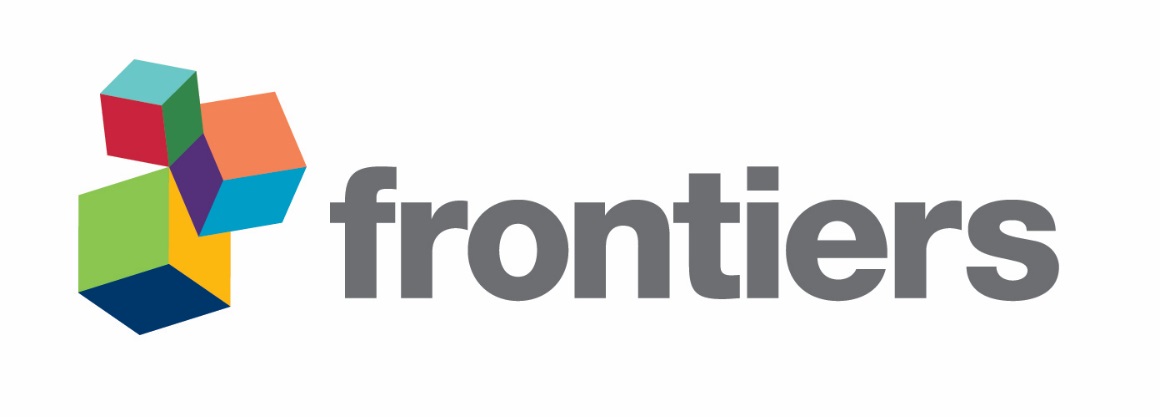
**
